# Adverse events in women giving birth in a labor ward: a retrospective record review study

**DOI:** 10.1186/s12913-021-07109-5

**Published:** 2021-10-14

**Authors:** Annika Skoogh, Marie Louise Hall-Lord, Carina Bååth, Ann-Kristin Sandin Bojö

**Affiliations:** 1 Department of Health Sciences, Faculty of Health, Science and Technology, Karlstad University, S-651 88 Karlstad, Sweden; 2 Department of Health Science, Faculty of Medicine and Health Sciences, Norwegian University of Science and Technology, Teknologivn. 22, 2815 Gjøvik, Norway; 3 Faculty of Health, Welfare and Organisation, Østfold University College, P.O. Box 700, 1757 Halden, Fredrikstad Norway

**Keywords:** Adverse events, Childbirth, Global trigger tool, Harm, Labor, Obstetric care, Patient safety, Record review

## Abstract

**Background:**

Childbirth could negatively affect the woman’s health through adverse events. To prevent adverse events and increase patient safety it is important to detect and learn from them. The aim of the study was to describe adverse events, including the preventability and severity of harm during planned vaginal births, in women giving birth in the labor ward.

**Methods:**

The study had a descriptive design with a retrospective birth record review to assess the preventability of adverse events using the Swedish version of the Global Trigger Tool. The setting was a labor ward in Sweden with low-risk and risk childbirths. Descriptive statistics, Pearson’s Chi-square test and Student’s t-test were used.

**Results:**

A total of 38 adverse events (12.2%) were identified in 311 reviewed birth records. Of these, 28 (73.7%) were assessed as preventable. Third- or fourth-degree lacerations and distended urinary bladder were most prevalent together with anesthesia-related adverse events. The majority of the adverse events were classified into the harm categories of ‘prolonged hospital care’ (63.2%) and ‘temporary harm’ (31.6%). No permanent harm were identified, but over two-thirds of the adverse events were assessed as preventable.

**Conclusions:**

This first study using Global Trigger Tool in a labor ward in Sweden identified a higher incidence of adverse events than previous studies in obstetric care. No permanent patient harm was found, but over two-thirds of the adverse events were assessed as preventable. The results draw particular attention to 3^rd^-or 4^th^-degree lacerations, distended urinary bladder and anesthesia-related adverse events. The feedback on identified adverse events should be used for systematic quality improvement and clinical recommendations how to prevent adverse events must be implemented.

**Supplementary Information:**

The online version contains supplementary material available at 10.1186/s12913-021-07109-5.

## Background

The ground-breaking report *To err is human* published in 2000 estimated that up to 98,000 patients die annually in hospitals due to unintentional human error, contributing to harm and adverse events [1]. Twenty years later, potentially preventable adverse events are still a major challenge, and improved patient safety is a key priority [2]. Childbirth is common in hospitals and is usually a positive experience for both the woman and her partner [3]. The midwives’ responsibility and goal is to protect and support women during birth [4]. The World Health Organization (WHO) [3] stresses the importance of evidence-based practice and antenatal care as contributors to a positive birth experience. However, childbirth could negatively affect the woman’s health through adverse events, harm, morbidity, suffering and even death [5–7]. Adverse events may also affect her capabilities to care for the newborn [8]. To prevent adverse events from occurring in connection to childbirth, it is important to detect these adverse events and learn from them [1]. A common definition of adverse events is ‘unintended physical injury resulting from or contributed to by medical care that requires additional monitoring, treatment or hospitalization, or that results in death’([9] p.5).

A systematic review demonstrated the incidence of adverse events in hospitals was 9.2%, with a preventability incidence of 43.5%. In obstetric care, the incidence was 5.9%, and in the labor and delivery room, the incidence was 3.7% [10]. Obstetric care has been included in studies of in-hospital adverse events [11], but sometimes these results examine obstetric care in combination with either gynecology [12, 13] or surgery [14, 15]; thus, rates of obstetric adverse events are difficult to pinpoint. Studies in obstetric care alone show that adverse event rates vary from 0.4–3.6%, with a preventability incidence of up to 56.3% [16–19]. Due to different review methods, samples, inclusion criteria, adverse event types and context of care, it is difficult to draw any conclusions from the results based on these studies.

Measuring harm can be done in different ways, and Vincent et al. [20] advocate for the measurement of past harm as one of five crucial dimensions for monitoring safety. They stress that to assess harm in healthcare, all kinds of events must be considered. Michel [21] found that the voluntary reporting of incidents underestimated adverse events, while record review was more effective. A common record review method is the Global Trigger Tool (GTT) [9], which has been found to be sensitive and reliable for detecting adverse events [22]. In obstetrics, various methods have been used for reporting adverse events, such as GTT [11], clinical surveillance [19], a combination of voluntary incident reports and quality indicators [18] and different screening guides [16, 17].

The GTT, from the Institute for Healthcare Improvement, was developed in 2003 [9] and is commonly used for tracking and measuring adverse events in healthcare [22]. The GTT has been adapted to the Swedish healthcare context [23, 24] and was implemented in the national safety program [25]. The GTT contains triggers organized in different modules, where one module concerns perinatal triggers. There are previous record review studies measuring adverse events in obstetric care, including adverse events in the labor ward. However, few studies have included only adverse events during planned vaginal births in the labor ward, and to our knowledge, there are no such studies in Sweden. The aim of this study was therefore to describe adverse events, including the preventability and severity of harm during planned vaginal births, in women giving birth in the labor ward.

## Methods

### Design

The study had a descriptive design with a retrospective birth record review to assess the preventability of adverse events using the Swedish version of the GTT.

### The global trigger tool

The Swedish adaption and translation of the GTT [23] was used for the review of birth records from admission to the labor ward and within 30 days of discharge. The Swedish version of the GTT includes guidelines for the assessment of adverse events in connection to each trigger [24]. The Swedish version of the tool contains 44 triggers organized into six modules. A trigger is a predefined ‘clue’, and the identification of a positive trigger in the record may indicate the presence of an adverse event. The six modules are Care module triggers (*n* = 18), Laboratory module triggers (*n* = 5), Surgical and other invasive procedures module triggers (*n* = 7), Medication module triggers (*n* = 3), Intensive care module triggers (=5) and Perinatal module triggers (*n* = 6). There are four types of adverse events: *Care*, *Infections*, *Complications in surgical and other invasive procedure* and *Other*. Each type of adverse event has several subtypes.

Assessment of preventability is described as a proactive patient safety perspective by the Patient Safety Law [26]. The assessment of preventability is therefore included in the Swedish version of the GTT [23, 24]. A 4-degree scale describes different levels of preventability: 1 = The adverse event was not preventable, 2 = The adverse event was probably not preventable, 3 = The adverse event was probably preventable, and 4 = The adverse event was preventable [23]. According to the previous study by Rutberg et al. [27], degrees 1 and 2 were grouped and contrasted with degrees 3 and 4. Only degrees 3 (probably preventable) and 4 (preventable) are termed preventable adverse events.

The severity of harm includes five categories labeled E-I, from ‘Temporary harm’ to ‘Contributed to patient’s death’ [28].

The variables of age, mode of birth, parity and gestational age were collected from the birth records.

### Setting and sample

The setting was a labor ward in a region in Sweden, with both low risk and risk childbirths, with approximately 2600 childbirths annually. The sample consisted of electronic birth records of women with planned vaginal births in the labor ward. Both women with low risk and risk childbirths were included.

The inclusion criteria were women ≥18 years registered in Sweden with singleton spontaneous vaginal birth, instrumental vaginal birth or emergency cesarean section [29, 30]. The exclusion criteria were women with multiple births (e.g., twins, triplets), elective cesarean section, unintentional birth outside of the hospital, or stillbirth [29, 30].

**Fig. 1:**
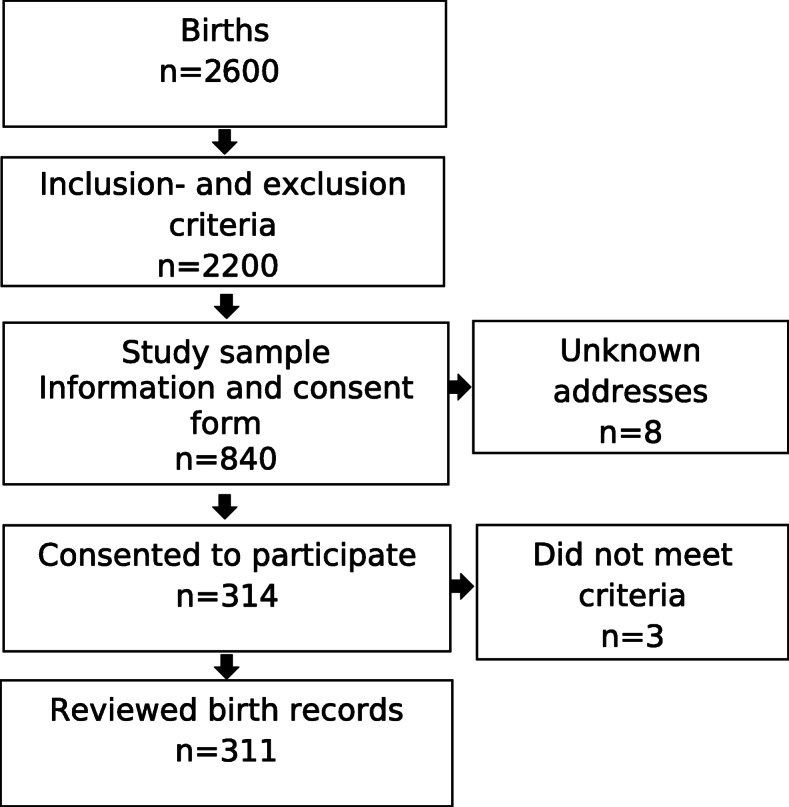
Flowchart of the study sample process

Based on the inclusion and exclusion criteria, the sample comprised of 2200 childbirths. The number of required birth records was estimated according to the incidence of 3^rd^- or 4^th^-degree lacerations. The motivation to choose the 3^rd^- or 4^th^-degree lacerations for the power analysis was based on the predefined perinatal adverse event in the GTT [23, 24], and has been annually reported from the labor wards in Sweden to the Swedish Medical Birth Register [31]. In 2013, the incidence of 3^rd^- or 4^th^-degree lacerations was 3.6% according to the Swedish Medical Birth Register [32]. To detect the incidence of 3.6% in the record review, 40 birth records per month for 14 months were needed. Since informed consent from the women to review their birth records was required, a loss of at least 30% was expected. Therefore, the number of birth records was increased to 60 per month, giving a total of 840 records required (see Fig. 1).

### Procedure

A civil servant in the region provided a simple random sampling of 60 birth records each month between January 2015 and February 2016. The first author received the list of the women’s names and addresses. Written information about the study and a request to participate in the study by giving consent for their birth record to be reviewed was sent to the women. The information was given to all women in Swedish, English and the two most common languages requiring interpretation needs in the labor ward, Arabic and Somali.

### Record review process

The birth record review took place in the labor ward between March 2016 and January 2017. Two of the authors (AS, AKSB) with clinical experience as midwives and knowledge of the context independently reviewed 11 birth records to test the trigger tool. The agreement between the reviewers was 100% for detecting the same positive triggers. The first author conducted the record review of the remaining birth records. The positive triggers and the identified potential adverse events were discussed by the research group. For validation, an obstetrician with previous experience in birth record review using the GTT also reviewed the birth records with identified adverse events. The adverse events, preventability and severity of harm were discussed and resulted in agreement.

### Statistics

IBM SPSS Software version 22 was used to analyze the data with descriptive statistics displaying frequencies, percentages, means and standard deviations. Subgroup analyses between women with no adverse events (*n* = 277) and women with adverse events (*n* = 34) were conducted. The Pearson’s Chi-square test was used for the analyses of the differences between the two groups on the following variables: mode of birth [spontaneous vaginal birth (*n* = 259), instrumental vaginal birth (*n* = 30), emergency cesarean section (*n* = 22)], parity [nulliparous (*n* = 151), parous (*n* = 160)] and gestational age [< 36 + 6 (*n* = 10), 37–41 (*n* = 289), ≥42 + 0 (*n* = 12)]. The Student’s t-test was run for the continuous variable age. A value of *p* < 0.05 was considered statistically significant.

## Results

In total, 311 (37.5%) of 829 women consented to have their birth records reviewed. Only five women consented in a foreign language (English *n* = 3, Arabic n = 1, Somali n = 1).

Eight women had unknown addresses and three women did not meet the inclusion criteria. The single reminder boosted participation by 92 women (11%) (Fig. 1). The women had a mean age of 31 years (SD 4.8) (Table 1). Women with spontaneous vaginal birth dominated, followed by instrumental vaginal birth and emergency cesarean section. Regarding parity, around half of the women were nulliparous (i.e. first-time mothers).

**Table 1:** Characteristics of the women (*n* = 311)

	n	%
**Age**		
< 25	23	7.4
25–35	220	70.7
> 35	68	21.9
**Mode of birth**		
Spontaneous vaginal birth	259	83.3
Instrumental vaginal birth	30	9.6
Emergency cesarean section	22	7.1
**Parity**		
Nulliparous	151	48.6
Parous	160	51.4
**Gestational age** (at childbirth)		
< 36 + 6	10	3.2
37–41	289	92.9
≥ 42 + 0	12	3.9

### Number and type of adverse events

In total, 209 positive triggers in 118 birth records were identified (see Additional file). No adverse events were identified in 277 (89.1%) of 311 birth records. In 34 (10.9%) birth records, adverse events were detected. Two adverse events were identified in four of the records, giving a total of 38 adverse events. The adverse events were classified into three of the four types (*Other*, *Care*, *Infections*) (Table 2).

**Table 2:** Adverse events in type, subtype and preventability

	Total adverse events *n* = 38	Preventable adverse events n = 28
***Other***		
**Postpartum adverse event/obstetric adverse event**		
3^rd^- or 4^th^-degree lacerations	10	9
Laceration (cervix/vagina)	5	0
Obstetric pelvic hematoma	2	0
**Anesthesia-related adverse event**		
Postdural puncture headache	3	3
Unintentional dural puncture	2	2
Unintentional long-term neurological impact after spinal anesthesia	1	1
**Neurological adverse event**		
Transient loss of sensation after positioning on operating room table	1	1
Transient loss of sensation after positioning leg support in labor room	1	1
**Other adverse events**		
Fracture of coccyx	1	0
***Care***		
**Distended urinary bladder**	7	7
***Infections***		
**Sepsis**	1	1
**Urinary tract infection**	2	1
**Infection other**		
Infection after repaired laceration	1	1
Fever in connection with prolonged premature rupture of membranes	1	1

In the type *Other*, 26 (68.4%) adverse events were identified and sorted into four subtypes: postpartum adverse event/obstetric adverse event, anesthesia-related adverse event, neurological adverse event and other adverse events. There were nine  3^rd^-degree lacerations and one 4^th^-degree laceration.

### Preventable adverse events and the severity of harm

Of the 38 adverse events, 28 (73.7%) were assessed to be preventable (Table 2). Nine of the ten 3^rd^- or 4^th^-degree lacerations, the anesthesia-related adverse events and the distended urinary bladder were assessed as preventable. The neurological adverse events derived from positioning were assessed as preventable. Four preventable adverse events involved infections. The severity of harm categories for the adverse events are presented in Table 3. The most prevalent categories were E and F.

**Table 3:** Severity of harm (*n* = 38)

Category	Definitions	n	%
E	Contributed to or resulted in temporary harm and required intervention	12	31.6
F	Contributed to or resulted in temporary harm required outpatient care, readmission or prolonged hospital care	24	63.2
G	Contributed to or caused permanent patient harm	0	0
H	Event that required lifesaving interventions required within 60 min	2	5.3
I	Contributed to patients death	0	0

### Subgroup analyses

Subgroup analyses were conducted between women with no adverse events (n = 277) and women with adverse events (n = 34) for age, gestational age, mode of birth and parity. There were significant differences regarding mode of birth and parity. Women with instrumental vaginal birth (30%) were more likely to sustain an adverse event than women with spontaneous vaginal birth (9.3%) or emergency cesarean section (4.5%) (*p* = 0.002). Furthermore, the proportion of nulliparous women (14.6%) who sustained an adverse event was higher than the proportion of parous women (7.5%) (*p* = 0.046).

## Discussion

The aim of this study was to describe adverse events, including the preventability and severity of harm during planned vaginal births, in women giving birth in the labor ward. The results of this first GTT study in planned vaginal births in Sweden provide important insights into adverse events and preventability. The results highlight adverse events of a distended urinary bladder and anesthesia-related adverse events, which have not been reported in previous GTT studies. The current results confirm previous research concerning the incidence of adverse events in hospital care, while the incidence is higher than in previous studies in obstetric care.

The results of this study found adverse events in approximately 11% of the birth records. However, while our numbers of adverse events are higher than in prior studies set in an obstetric context [16–19], they are consistent with the numbers presented in the general hospital review of de Vries et al. [10], the scoping review of Schwendimann et al. [33] and the Swedish report of adverse events in obstetric care and gynecology [34]. The preventability incidence is in line with that of the first Swedish measurement of adverse events of 70% [35], but it is higher than that of the national report five years later, which reported a general preventability rate of 62% [13]. The rates for obstetric care combined with gynecology were 48% [34].

Concerning severity, most of the adverse events were assessed in categories E and F, namely, ‘prolonged hospital care’ and ‘temporary harm’, which is consistent with the findings of other studies [12, 14]. There were two adverse events assessed as serious category H, one of which was related to maternal sepsis. Knight et al. [6] reported that the incidence of this life-threatening condition has declined across the United Kingdom as an effect of raised awareness and the Global Maternal Sepsis Awareness campaign. A distended urinary bladder was assessed in category F in all instances, resulting in prolonged hospital care but no permanent harm.

A distended urinary bladder is common in other care contexts [13, 36]. Joelsson-Alm et al. [37] interviewed mothers who described micturition problems after developing a distended urinary bladder. They described restrictions on everyday life and feelings of guilt because they were unable to care for their newborns properly [37]. WHO has recommended that urine void should be documented within six hours [3], not explicit how to prevent a distended urinary bladder postpartum. It is important to develop and implement recommendations to prevent distended urinary bladder during childbirth.

In the present study, ten women (3.2%) sustained 3^rd^- or 4^th^-degree lacerations, which is in line with the rate of 3.1% reported in the 2015 Swedish Medical Birth Register [38, 39]. In nine of ten records, 3^rd^- or 4^th^-degree lacerations were assessed as preventable. No documentation of perineal protection was found in these nine records, although perineal protection is within the midwives’ competence and should be documented [40]. Perineal protection (limited to ‘hands-on’) is one recommended intervention to prevent 3^rd^- or 4^th^-degree lacerations [24]. Other interventions to prevent 3^rd^- or 4^th^-degree lacerations are e.g., risk assessment, warm perineal compresses and delivery positioning during childbirths [41].

The subgroup analysis in this study indicated that the proportion of nulliparous women who sustained an adverse event was greater than the proportion of parous women. Being a first-time mother has been shown to be related to an increased risk for obstetrical interventions (e.g., instrumental vaginal birth and emergency cesarean section) [42]. Obstetrical interventions in childbirths could be lifesaving, but unnecessary interventions could lead to adverse events for women [8].

Previous studies have found that women who sustained severe perineal lacerations suffered physically and psychologically and were not satisfied with their healthcare support [7, 43]. Professional support in terms of a midwife being present in the labor room is important to create safe conditions during childbirth and can lead to decreased anxiety and tension in women [44]. Supportive care includes emotional support, information, advice about coping practices and comfort procedures. Support enhances physiological labor processes and may reduce obstetrical interventions [45].

It is important that midwives be given organizational prerequisites to practice a watchful attendance during childbirth [4]. This notion is supported by the Swedish website *Pelvic floor education.se*, which recommends the assistance of two midwives during the end of the childbirth [41]. Both international and national clinical recommendations to prevent obstetrical lacerations have been developed [41, 46, 47]. It is important to educate healthcare professionals in labor wards and implement those recommendations in clinical practice.

We identified three instances of postdural puncture headache within anesthesia-related adverse events. It has been considered a benign headache, but a review highlighted that headache and backache may persist after unintentional dural puncture for more than six weeks [48].

In the Care module triggers, see Additional file, we identified 35 positive triggers that were placed in the trigger Other*.* The most common trigger among these 35 triggers was Hemorrhage > 1000 ml (*n* = 17). In the original version of the GTT [9], a trigger labeled ‘Estimated blood loss greater than 500 ml for vaginal delivery, or greater than 1,000 ml for cesarean delivery’ was included, but this trigger was removed from the Swedish version. In Sweden, normal postpartum hemorrhage is defined as no more than 1000 ml [30]. The WHO states that postpartum hemorrhage is commonly defined as a blood loss of 500 ml or more within 24 h after birth [49]. It is important for the midwife to pay attention to the risk of abnormal hemorrhage which may lead to harm. Further development and modification of the GTT in relation to obstetric care may be needed. The GTT was originally developed for adult hospital inpatients. Thereafter, trigger tools to detect adverse events have been adapted and modified for hospital specialties such as pediatric care [50] and home healthcare [36]; similar adaption and modification could be applied to obstetric care.

### Limitations

Some limitations of this study must be addressed. The study was conducted at a single site, and fewer women than expected consented to participate. The research ethical board required that women consent to having their records reviewed. A low proportion of returned consent forms might be explained by barriers identified in the review, according to van der Zande et al. [51], who described pregnant women’s reasons not to participate in research as related to inconvenience, risks, medical reasons and third-party influences. Despite translated research information, few consent forms in the translated languages were returned. The women in our study were representative of the population captured in the Swedish Medical Birth Register in 2015 related to 3^rd^- or 4^th^-degree lacerations (3.1%), spontaneous vaginal birth (83.1%) and emergency cesarean section (7.8%), whereas the rate of instrumental vaginal birth (7.1%) was slightly higher in the present study [39]. Regarding parity, the number of nulliparous women in our study was slightly higher than that noted in the population (43.8%) [39]. It should be noted that the target number of birth records based on the power analysis was not reached, and the low numbers may be the reason for the limited number of significant differences.

The birth record review was mainly carried out by the first author with knowledge and experience of the context. Furthermore, the research group and the obstetrician were involved in the review process. The obstetrician and the first author agreed on all adverse events. Despite the thorough birth record review, the number of adverse events might have been underestimated. Another reason for the underestimation of adverse events may depend on the information and quality of the documentation [52]. Electronic information was always available and mostly informative, although not always complete. Therefore, adverse events might have been missed given that the midwives and physicians did not always document all care, such as perineal protection.

## Conclusion

This first GTT study identified a higher incidence of adverse events in planned vaginal births in a labor ward in Sweden than previous studies in obstetric care. Over two-thirds of the adverse events were assessed as preventable. The results draw particular attention to distended urinary bladders, 3^rd^- or 4^th^-degree lacerations, and anesthesia-related adverse events. Healthcare professionals and clinical leaders can use the information about the identified adverse events for quality improvement in the labor ward. It is crucial to use a systematic approach to quality improvement, such as plan-do-study-act (PDSA) cycles, to monitor and learn about adverse events. Additional studies are needed to increase the knowledge of adverse events in connection to childbirth.

## Supplementary Information

**Additional file 1.**

## Data Availability

The datasets used and/or analyzed during the current study are available from the corresponding author on reasonable request.

## Abbreviations

GTT: Global Trigger Tool
WHO: World Health Organization
PDSA: plan-do-study-act
