# Supplementary material for: Adverse events in women giving birth in a labor ward: a retrospective record review study
Source: BMC Health Serv Res. 2021 Oct 14;21:1093. doi: 10.1186/s12913-021-07109-5 (PMC8518258; doi:10.1186/s12913-021-07109-5)
Supplement: Supplementary file 1 — Additional file 1. [file 12913_2021_7109_MOESM1_ESM.docx]

**Additional file**

| Modules with triggers | Total  n=209 | Specified Other  n=35 |
| --- | --- | --- |
| **Care module triggers**  Transfusion or use of blood products   Cardiac arrest or abnormal vital signs   Distended urinary bladder   Neurological adverse event  Positive blood culture   Healthcare-associated infection  Transfer to higher level of care   Acute consultation within 2 days of discharge  Readmission within 30 days  Documentation of mistake   Other  Hemorrhage >1000 ml  Manual removal of the placenta after vaginal birth  Laceration (cervix/vagina)  Obstetric pelvic hematoma  Fracture of coccyx  **Laboratory module triggers**  Low hemoglobin level  Abnormal sodium level  **Surgical and other invasive procedures module triggers**  Anesthesia***-***related adverse event **Intensive care module triggers**  Treatment in intensive care unit **Perinatal module triggers**  Reduced vitality of newborn  Transfer of mother/newborn  Terbutaline use  3^rd^- or 4^th^-degree lacerations  Induction of labor  Instrumental vaginal birth | 14  2 7  3 1 7  1 1 14  3  35  23  1  6  1  2  16  18  10  14  30 | 17  10  5  2  1 |

Positive triggers
